# *Clostridium perfringens* epsilon toxin vaccine candidate lacking toxicity to cells expressing myelin and lymphocyte protein

**DOI:** 10.1038/s41541-019-0128-2

**Published:** 2019-07-30

**Authors:** Helen Morcrette, Monika Bokori-Brown, Stephanie Ong, Leo Bennett, Brendan W. Wren, Nick Lewis, Richard W. Titball

**Affiliations:** 1 University of Exeter, Exeter, EX4 4QD UK; 2 Department of Pathogen Molecular Biology, London School of Hygiene and Tropical Medicine, London, UK; 3 One Health Ventures Ltd, 23 Bewley Street, London, SW19 1XF UK

**Keywords:** Protein vaccines, Bacterial infection

## Abstract

A variant form of *Clostridium perfringens* epsilon toxin (Y30A-Y196A) with mutations, which shows reduced binding to Madin–Darby canine kidney (MDCK) cells and reduced toxicity in mice, has been proposed as the next-generation enterotoxaemia vaccine. Here we show that, unexpectedly, the Y30A-Y196A variant does not show a reduction in toxicity towards Chinese hamster ovary (CHO) cells engineered to express the putative receptor for the toxin (myelin and lymphocyte protein; MAL). The further addition of mutations to residues in a second putative receptor binding site of the Y30A-Y196A variant further reduces toxicity, and we selected Y30A-Y196A-A168F for further study. Compared to Y30A-Y196A, Y30A-Y196A-A168F showed more than a 3-fold reduction in toxicity towards MDCK cells, more than a 4-fold reduction in toxicity towards mice and at least 200-fold reduction in toxicity towards CHO cells expressing sheep MAL. The immunisation of rabbits or sheep with Y30A-Y196A-A168F induced high levels of neutralising antibodies against epsilon toxin, which persisted for at least 1 year. Y30A-Y196A-A168F is a candidate for development as a next-generation enterotoxaemia vaccine.

## Genetic toxoids avoid MAL for reduced host toxicity

Cells expressing myelin and lymphocyte protein (MAL), the putative receptor for Clostridium perfringens’ epsilon toxin, can be sensitive to otherwise attenuated mutants of the toxin. Here, the team led by Richard Titball at United Kingdom’s University of Exeter found that a previous variant exhibits differential toxic effects when cells express sheep or human MAL. To circumvent this, Titball’s team applied site-directed mutagenesis of the receptor binding site to develop a new variant with enhanced reduction in toxicity towards MAL-expressing cells and able to induce high levels of neutralising antibodies upon immunisation of sheep. These findings suggests that testing genetic toxoids in cells expressing MAL from the target species might be relevant for enterotoxaemia vaccine development and warrant further studies into the role of MAL in epsilon toxin-mediated pathogenesis.

## Introduction

Epsilon toxin is one of the major toxins produced by *Clostridium perfringens*.^1^ The toxin is produced by the bacterium as a prototoxin, which is activated by proteolysis, with the consequent release of carboxy-terminal and amino-terminal peptides from the protein.^2–4^ This toxin is important in the field of veterinary medicine because it plays a major role in the development of enterotoxaemia of sheep and goats, and occasionally of other livestock animals. The toxin is produced in the gut of these animals, often following the sudden ingestion of feedstuffs rich in carbohydrates.^5,6^ By an unknown mechanism(s), the toxin crosses the gut wall and enters the blood stream, becoming preferentially targeted to the kidney, heart, lungs, and brain, and causing oedema. The disease is often called pulpy kidney, although lesions in the kidneys of affected animals are not observed. Effects on the central nervous system result in neurological symptoms,^7^ and in many animals the disease is rapidly fatal. Consequently, the disease is of global economic significance. More recently, evidence has been presented that epsilon toxin may play a role in the development of multiple sclerosis in humans.^8,9^

The toxin acts by binding to host cells, and there is evidence that seven monomers of the protein assemble into a pore, which spans the cell membrane,^10^ resulting in unregulated ion movement across the membrane and cell death.^11^ The identity of the cell surface receptor for the toxin is still not fully clarified. However, there is good evidence that the toxin binds to myelin and lymphocyte protein (MAL).^12,13^ CHO cells, which are normally highly resistant to the toxin, become sensitive when expressing MAL,^12,13^ and MAL knockout mice are reported to be highly resistant to the toxin.^12^ Other proteins that have been shown to play a role in epsilon toxin binding may play accessory roles in toxin binding.^14–16^

A number of commercial vaccines are available for the prevention of enterotoxaemia. They are typically produced by treating a *C. perfringens* culture filtrate with formaldehyde, resulting in detoxification of epsilon toxin. These vaccines contain proteins in addition to epsilon toxin, and there can be considerable batch-to-batch variation in the immunogenicity of these preparations. Inflammatory responses following vaccination have been reported to result in reduced feed consumption. These shortfalls have prompted work to devise improved vaccines, and a number of recombinant immunogens have been reported, including formaldehyde-treated epsilon toxin produced from *Escherichia coli*^17^ and site-directed mutants (genetic toxoids) of epsilon toxin with reduced toxicity.^11,18–22^ Site-directed mutants offer the advantage that chemical detoxification, a process that can result in batch-to-batch variation in immunogenicity, is avoided. However, the high potency of the toxin can make it difficult to completely abolish toxicity. The toxicity of the mutants has, until now, been assessed using either Madin–Darby canine kidney (MDCK) cell cultures or in mice.

We have previously constructed and tested a site-directed mutant of epsilon toxin (Y30A-Y196A).^20^ This mutant had >430-fold decrease in cytotoxicity towards MDCK cells compared with the wild-type toxin and showed reduced toxicity in mice. In this study, we report that unexpectedly this mutant showed only marginal changes in toxicity towards CHO cells expressing sheep or human MAL (hMAL). We have therefore set out to identify mutations that could be incorporated into the Y30A-Y196A mutant to reduce toxicity towards CHO cells expressing MAL. We have tested these mutants in MDCK.2 cells and in CHO cells expressing MAL in mice and in sheep. These mutants are promising genetic toxoids for incorporation into next-generation enterotoxaemia vaccines.

## Results

### Mutagenesis of residues flanking the β-octyl-glucoside binding site

**Fig. 1:**
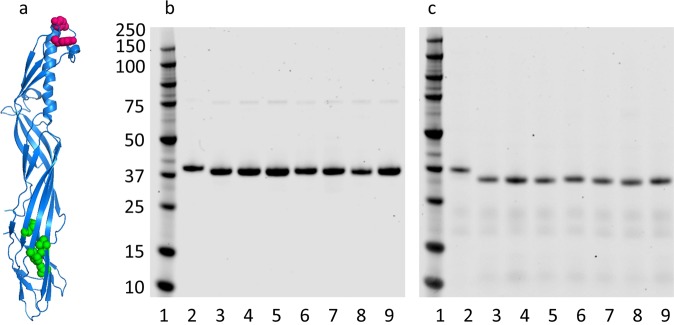
Variant proteins tested in this study. **a** The location of residues previously mutated (Y30 and Y196; ref. ^20^) are shown in red and the location of residues in the β-octyl-glucoside binding cleft (V72, F92, H149, V166, A168; ref. ^19^) mutated in this study are shown in green. Purified proteins were separated by sodium dodecyl sulfate-polyacrylamide gel electrophoresis (SDS-PAGE) and visualised by Coomassie staining before **b** and after **c** activation with trypsin. Lane 1 = molecular weight marker (kDa); 2 = wild-type toxin; 3 = Y30A-Y196A; 4 = Y30A-Y196A-H149A; 5 = Y30A-Y196A-A168F; 6 = Y30A-Y196A-F92A; 7 = Y30A-Y196A-V166A; 8 = Y30A-Y196A-V72F; 9 = Y30A-Y196A-H149A-A168F. Gels shown derive from the same experiment and were processed in parallel

In a previous study, we identified a glycan (β-octyl-glucoside) binding site in domain 3 of epsilon toxin, and suggested that this site may be a second receptor binding site.^19^ Here we selected five residues within or flanking this site for mutagenesis (V72, F92, H149, V166, A168; Fig. 1a) to evaluate their role in toxicity. Using a plasmid template, which encoded the Y30A-Y196A variant form of epsilon prototoxin,^20^ residues V72, F92, H149, V166 or A168 were mutated to alanine (F92, H149, V166) or phenylalanine (V72, A168) and the His-tagged proteins encoded by the mutated genes were expressed in *E. coli* and purified (Fig. 1b). We also expressed and purified the Y30A-Y196A variant form of epsilon toxin and a mutant, which contained both H149A and A168F mutations, in addition to Y30A-Y196A. The authenticity of the proteins was verified in two ways. First, the genes encoding the mutant genes were sequenced to validate the presence of the expected mutation. Second, the purified proteins were analysed by mass spectrometry to confirm that the experimentally determined mass matched the expected molecular mass of the protein. For studies in mice, rabbits or sheep, the proteins had endotoxin levels below 40 endotoxin units/ml.

### Thermostability of proteins

The structural stabilities of the wild-type epsilon toxin, the Y30A-Y196A variant protein and the six mutants of Y30A-Y196A were assessed using thermostability assays. This revealed that the melting temperature (*T*_m_ in °C) of the Y30A-Y196A variant was lower than that of wild-type toxin. However, the introduction of additional mutations (V72F, F92A, H149A, V166A, A168F) into the Y30A-Y196A variant protein only resulted in small changes in the thermostability of the proteins (Table 1), indicating that these substitutions did not destabilise the tertiary structures of the protein. The Y30A-Y196A-H149A-A168F had the lowest *T*_m_, indicating that this was the least stable of the mutants tested (Table 1).

**Table 1:** Melting temperature (*T*_m_) of wild-type and variant epsilon toxins

Protein	Mean *T*_m_ (°C) ± SD
Wild-type epsilon toxin	67.4 ± 1.45
Y30A-Y196A	60.6 ± 0.15
Y30A-Y196-A168F	62.0 ± 0.22
Y30A-Y196A-H149A	60.9 ± 2.28
Y30A-Y196A-V166A	59.9 ± 0.47
Y30A-Y196A-V72F	59.5 ± 0.10
Y30A-Y196A-F92A	58.2 ± 0.09
Y30A-Y196A-H149A-A168F	57.4 ± 0.02

Thermostability of proteins was determined by the Boltzmann method using the Protein Thermal Shift software (Applied Biosystems). Results represent the mean and standard deviation (SD) of measurements from duplicate samples

### Toxicity of the variant proteins in cell culture

The trypsin-activated purified epsilon toxin proteins were tested for toxicity towards MDCK.2 cells and CHO cells expressing human MAL (hMAL) or sheep MAL (sMAL) (Table 2). The Y30A-Y196A mutations resulted in over 165-fold reduction in toxicity towards MDCK cells compared to the wild type. However, this mutant showed 2.3-fold increased toxicity towards CHO cells expressing hMAL and an 8.6-fold reduction in toxicity towards CHO cells expressing sMAL, compared with wild-type toxin. Of the mutants tested, the additional H149A or A168F mutations introduced into Y30A-Y196A resulted in the most marked reductions in toxicity towards CHO cells expressing hMAL or sMAL (Table 2).

**Table 2:** Toxicities of the variant proteins tested in this study

Protein	Toxicity towards MDCK cells	Lethality (activated epsilon toxin) in mice (i.p.)	Lethality (non-activated epsilon toxin) in mice (s.c.)	Toxicity towards CHO-hMAL cells	Toxicity towards CHO-sMAL cells
Wild-type toxin	9.7 ± 1.2 nM	20^a^–200 ng^b^	ND	12.7 ± 0.68 nM	1.6 ± 0.5 nM
Y30AY-196A	1.6 ± 0.14 µM	2^a^–20 µg^b^	ND	5.5 ± 0.43 nM	13.75 ± 0.98 nM
Y30AY-196A-H149A	>6 µM	2^a^–20 µg^b^	ND	>2.5 µM	>2 µM
Y30AY-196A-A168F	>6 µM	20^a^–200 µg^b^	>200 µg^a^	>2.5 µM	>3 µM
Y30AY-196A-F92A	2.76 ± 0.18 µM	ND	ND	9.94 ± 1.35 nM	47.6 ± 13 nM
Y30AY-196A-V166A	>3 µM	ND	ND	14.3 ± 0.98 nM	76.3 ± 11 nM
Y30AY-196A-V72F	<3 µM	<2 µg^b^	ND	>3 µM	ND
Y30AY-196A-H149A-A168F	>6 µM	>20 µg^a^	ND	>3 µM	>3 µM

Toxicities towards MDCK cells, CHO-hMAL cells and CHO-sMAL cells are the means of three replicates with standard error of the mean (SEM) values shown

*MDCK* Madin–Darby canine kidney, *CHO* Chinese hamster ovary, hMAL human MAL, *sMAL* sheep myelin and lymphocyte protein, *ND* not determined

^a^All mice (*n* = 6) survived at this dose

^b^All mice (*n* = 6) reached a humane end point at this dose

### Toxicity of the variant proteins in mice

The variant proteins that showed the greatest reduction in toxicity towards CHO-hMAL cell cultures (Y30A-Y196A-H149A, Y30A-Y196A-A168F and Y30A-Y196A-H149A-A168F) were next tested for toxicity in mice (Table 2). When given by the intaperitoneal (i.p.) route, the MLD dose of trypsin-activated wild-type toxin was between 20 and 200 ng. Compared to the wild type, the MLD doses of trypsin-activated Y30A-Y196A or Y30A-Y196A-H149A were reduced 100–1000-fold. The MLD doses of trypsin-activated Y30A-Y196A-A168F or Y30A-Y196A-H149A-A168F were above the highest dose tested (20 µg). Without prior activation with trypsin, the MLD dose was more than 200 µg per mouse. The Y30A-Y196A-A168F protein was selected for further testing of toxicity by the subcutaneous (s.c.) route, either before or after trypsin activation.

### Antibody responses to the variant proteins

**Fig. 2:**
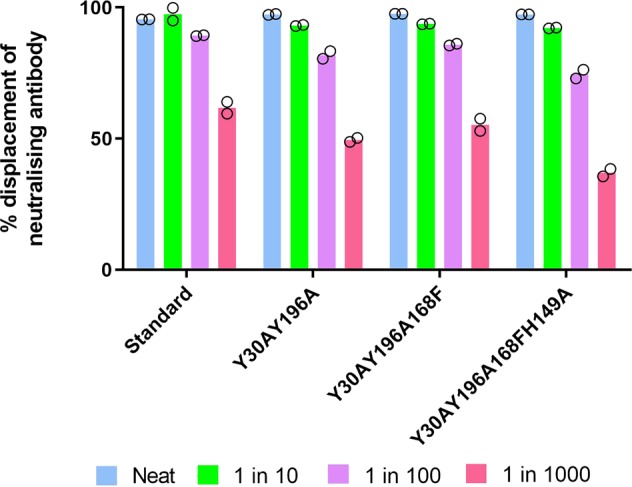
Ability of World Health Organisation (WHO) standard antitoxin (5 IU/ml) or rabbit sera raised against genetic toxoids to displace binding of a neutralising monoclonal antibody in a competitive enzyme-linked immunosorbent assay (ELISA). The sera were diluted as indicated in the legend before testing. Bars show the mean of two assays

Groups of three rabbits were immunised with the Y30A-Y196A, Y30A-Y196A-A168F or Y30A-Y196A-H149A-A168F genetic toxoids given with Freund’s adjuvants. One week after the fourth immunising dose, serum was tested using a competition enzyme-linked immunosorbent assay (ELISA) and using an MDCK neutralisation assay for antibodies against epsilon toxin. In both assays, we included World Health Organisation (WHO) International Standard epsilon horse antitoxin serum. All of the rabbits developed antibodies that reacted with wild-type epsilon toxin when tested using competition ELISA. The titres were broadly similar in the sera from individual rabbits in each immunisation group, and therefore we pooled the sera for subsequent tests (Fig. 2).

We also tested the sera for ability to neutralise toxicity towards MDCK cells, using an assay previously shown to correlate with the mouse neutralisation test.^23–27^ We used WHO International Standard epsilon horse antitoxin serum, with a known neutralising antibody titre, to calibrate our assay. The pooled rabbit sera contained 21 IU/ml (international units/ml) of neutralising antibody.

### Immunisation of lambs

We selected the Y30A-Y196A-A168F protein for testing in sheep because it had induced a robust antibody response in rabbits, showed low toxicity in mice, and had a thermostability profile most similar to the wild-type protein. We immunised groups of five or six lambs with two doses of Y30A-Y196A-A168F prototoxin given with either Montanide ISA 61VG or aluminium hydroxide gel (alhydrogel) adjuvant (Table 3). One additional group of lambs had pre-existing antibodies against epsilon toxin at the start of the study, detected using Western blotting, and these lambs were immunised with two doses of Y30A-Y196A-A168F protein with Montanide ISA 61VG adjuvant. The sera from these animals and control sera from lambs, which had not been immunised, were tested for the presence of antibody able to neutralise epsilon toxin using three different assays. In all of these assays, we included dilutions of standardised sera containing a known concentration of neutralising antibodies, expressed as IU/ml.

**Table 3:** Neutralising antibody in pooled sera from lambs immunised with Y30A-Y196A-A168F

Treatment group	*n*		Neutralising antibody (IU/ml) measured using MDCK cells	Neutralising antibody (IU/ml) measured using CHO-sMaL cells	Neutralising antibody (IU/ml) measured using competition ELISA assay						
		Week post immunisation	13	13	3	7	13	16	25	38	52
Control	5		0	0	ND	ND	0	ND	ND	ND	ND
Y30A-Y196A-A168F + montanide ISA 61VG adjuvant	6		73 ± 2.4	154 ± 1.4	107 ± 7	311 ± 10	231 ± 6	207 ± 4	146 ± 12	85 ± 3	68 ± 3.6
Y30A-Y196A-A168F + alhydrogel adjuvant	5		0	0	ND	ND	<6^a^	ND	ND	ND	ND
Y30A-Y196A-A168F + montanide ISA 61VG adjuvant (lambs with pre-existing antibodies to epsilon toxin)	4		21 ± 0.1	40 ± 2.8	ND	ND	69 ± 6	ND	ND	ND	ND

Results shown at each time are the mean of duplicate assays, each with pooled sera from all animals (*n*) in that experimental group, ±SD

*MDCK* Madin–Darby canine kidney, *CHO* Chinese hamster ovary, *sMAL* sheep myelin and lymphocyte protein, *ND* not determined, *ELISA* enzyme-linked immunosorbent assay

^a^Fifty per cent displacement of neutralising antibody could not be achieved at the lowest dilution of sera tested

The results obtained using an MDCK.2 assay have previously been shown to correlate with the mouse neutralisation test.^23,24^ Using this assay we found that at week 13 post immunisation, the pooled sera from lambs immunised with toxoid in Montanide ISA 61VG adjuvant contained 73 IU/ml of neutralising antibodies (Table 3). In lambs that had pre-existing antibody and were immunised with toxoid in Montanide ISA 61VG, the level of neutralising antibodies was lower (21 IU/ml). We did not detect antibody in the sera from control lambs or in sera from lambs immunised with toxoid in alhydrogel using this assay.

We found a similar pattern of results using the competition ELISA or using an assay where we measured neutralisation towards CHO-sMAL cells, although the measured levels of neutralising antibody were higher using these assays than using the MDCK.2 cell suspension assay (Table 3). Also, using the competition ELISA we detected low levels of antibodies in the sera from lambs immunised with toxoid in alhydrogel. We also used the competition ELISA to measure the relative levels of antibodies up to 12 months after immunisation. At 52 weeks post immunisation, we were able to detect high levels of neutralising antibodies (Table 3).

## Discussion

In a previous study, we investigated the vaccine potential of a site-directed mutant of epsilon toxin with mutations in the putative receptor-binding domain (domain 1; ref. ^20^). We showed that a combination of Y30A and Y196A mutations markedly reduced the ability of the toxin to bind to and kill MDCK cells. We also showed that Y30A-Y196A had reduced toxicity in mice, suggesting that Y30A-Y196A mutant could form the basis of an improved recombinant vaccine against enterotoxaemia (Table 4). Polyclonal antibody raised against Y30A-Y196A provided protection against wild-type toxin in an in vitro neutralisation assay.

**Table 4:** Primers used for site-directed mutagenesis

Change	Primer sequence^a^
Y30A	Primer ‘Y43A_Forward’: GAAAGGAAGATATAATACAAAATATAATTACTTAAAGAGAATGGAAAAATAT**GCG**CCTAATGCTATGGCATATTTTGATAAGG Primer ‘Y43A_Reverse’: CCTTATCAAAATATGCCATAGCATTAGG**CGC**ATATTTTTCCATTCTCTTTAAGTAATTATATTTTGTATTATATCTTCCTTTC
Y196A	Primer ‘Y209A_Forward’: GTGAATGGGGAGAGATACCTAGT**GCG**TTAGCTTTTCCTAGGGATGGTTA Primer ‘Y209A_Reverse’: TAACCATCCCTAGGAAAAGCTAA**CGC**ACTAGGTATCTCTCCCCATTCAC
H149A	Primer ‘H149A_Forward’: CAAATACAAATACAAATACTAATTCAAAAGAAATTACT**GCT**AATGTCCCTTCACAAGATATACTA Primer ‘H149A_Reverse’: TAGTATATCTTGTGAAGGGACATT**AGC**AGTAATTTCTTTTGAATTAGTATTTGTATTTGTATTTG
V72F	Primer ‘V72F_Forward’: AGAACCATCAATGAATTATCTTGAAGATGTTTAT**TTT**GGAAAAGCTCTCTTAAC Primer ‘V72F_Reverse’: GTTAAGAGAGCTTTTCC**AAA**ATAAACATCTTCAAGATAATTCATTGATGGTTCT
F92A	Primer ‘F92A_forward’: TCTTAACTAATGATACTCAACAAGAACAAAAATTAAAATCACAATCA**GCG**ACTTGTAAAAATACTGATACAGTAAC Primer ‘F92A_reverse’: GTTACTGTATCAGTATTTTTACAAGT**CGC**TGATTGTGATTTTAATTTTTGTTCTTGTTGAGTATCATTAGTTAAGA
V166A	Primer ‘V166A_forward’: ATACTAGTACCAGCTAATACTACTGTAGAA**GCG**ATAGCATATTTAAAAAAAGTTAATGTTAAAG Primer ‘V166A_Reverse’: CTTTAACATTAACTTTTTTTAAATATGCTAT**CGC**TTCTACAGTAGTATTAGCTGGTACTAGTAT
A168F	Primer ‘A168F_Forward’: GATATACTAGTACCAGCTAATACTACTGTAGAAGTAATA**TTT**TATTTAAAAAAAGTTAATGTTAAAGGAAATGTAAAGTTAG Primer ‘A168F_reverse’: CTAACTTTACATTTCCTTTAACATTAACTTTTTTTAAATA**AAA**TATTACTTCTACAGTAGTATTAGCTGGTACTAGTATATC

^a^Bold bases indicate the codons used for substitution. All primer sequences are shown in 5′ to 3′ orientation

However, our previous study used the MDCK cell line to measure cytotoxicity, and subsequently it has been shown that CHO cell expressing MAL are also highly sensitive to the toxin.^12,13^ The possibility that MAL is a receptor for the toxin is supported by the finding that MAL-knockout mice become resistant to the effects of epsilon toxin^12^ and by the finding that the toxin can be isolated from cells complexed with MAL.^13^ In this study we report that the Y30A-Y196A mutant is marginally less toxic than wild-type toxin towards CHO cells expressing sMAL, but is more toxic to CHO cells expressing hMAL compared to wild-type epsilon toxin. This finding suggests that MAL from different species interacts differently with epsilon toxin. Our findings indicate that CHO cells, which express MAL from the target species to be immunised, should be used alongside MDCK cells and mice to assess the toxicity of variant forms of epsilon toxin. Our findings also suggest that toxicities of epsilon toxin variants in mice may not fully reflect their likely toxicities in sheep.

In this study, we introduced additional mutations to reduce the toxicity of Y30A-Y196A towards CHO cells expressing MAL. We have introduced these mutants into a region in domain 3 that has been implicated in sugar binding.^19^ A number of these mutants showed reduced toxicity in CHO-MAL cell cultures, and also towards MDCK cells and in mice. While our data do not confirm the identity of a second receptor binding site, they do confirm the role of this region in toxicity. We were able to produce all of these proteins, and on the basis of thermostability measurement, they did not show major changes in stability, suggesting that the conformation of the proteins was broadly similar to that of the wild-type epsilon toxin.

In this study, we measured neutralising antibody using three tests that avoid the use of mice. One of these tests, using MDCK cells, has previously shown to provide an accurate indication of the neutralising antibody titre when tested in mice, and is therefore an alternative to the mouse lethality test.^23–27^ The competitive ELISA and CHO-sMAL cell culture neutralisation test also revealed the presence of neutralising antibodies to epsilon toxin in sheep and rabbit sera. Livestock vaccines containing aluminium hydroxide or saponin as adjuvants often induce short-lived antibody responses.^28^ This necessitates boosting at intervals, sometimes as short as 4 months apart. Montanide ISA 61VG is a new ready-to-use mineral oil-based adjuvant for use in livestock, which offers the potential to induce high-level and long-lasting responses in animals.^28^ Our finding that the use of Montanide ISA 61VG adjuvant resulted in the induction of better antibody responses compared to the use of an alhydrogel adjuvant is similar to previous finding with a foot and mouth disease vaccine.^28^ A previous report has also shown that ISA 61VG is superior to ISA 201VG (water-in-oil) adjuvant or Montanide Gel 01 (aqueous polymer) adjuvant for the induction of antibody responses.^29^ We did not see any evidence of local side effects after using Montanide ISA 61VG adjuvanted protein in lambs, although others have reported local reactions using this adjuvant.^29^

For licensing of epsilon-toxoid vaccine in Europe, compliance with the European Pharmacopoeia (Ph. Eur.) monograph 0363 on *Clostridium perfringens* vaccines for veterinary use^30^ would be required. Based on our experimental data we can assess the likelihood that our Y30A-Y196A-A168F candidate, adjuvanted with Montanide ISA 61VG, would meet these requirements. European Pharmacopoeia monograph 0363 requires that mice dosed s.c. with 0.5 ml of the vaccine do not show signs of intoxication. We did not detect any sign of toxicity when 200 µg of Y30A-Y196A-A168F prototoxin was given s.c. to mice, indicating that our vaccine would comfortably meet the requirement for residual toxicity. In our studies, lambs were immunised with 200 µg of toxoid in a total volume of 3 ml. This volume, which is consistent with the doses of existing commercial vaccines given to sheep, would indicate a dose of 33 µg of toxoid in 0.5 ml of vaccine.

European Pharmacopoeia monograph 0363 also requires that rabbits given two minimum doses of vaccines 21–28 days apart develop neutralising antibody levels of at least 5 IU/ml. We did not test Y30A-Y196A-A168F adjuvanted with Montanide ISA 61VG in rabbits. However, the level of neutralising antibodies we elicited in sheep immunised with two doses of Y30A-Y196A-A168F prototoxin adjuvanted with alhydrogel were low. Previously neutralising antibody levels in the range 8–26 IU/ml have been reported after immunisation of rabbits or sheep with two doses of 200 µg of recombinant epsilon toxin, or recombinant *E. coli* expressing epsilon toxin, treated with formaldehyde given with aluminium-based adjuvants.^17,31–33^ These titres are broadly similar to the titres reported in sheep (up to 15 IU/ml; refs ^33,34^) or in goats (0.22–1.52 IU/ml; refs ^35,36^) vaccinated with commercially available vaccines. The neutralising antibody titres that we have found in sheep dosed with our toxoid with Montanide ISA 61VG adjuvant are well in excess of the reported minimum protective titres in sheep (0.1–0.3 IU/ml; refs ^34,35^) or in goats (1 IU/ml; refs ^35,36^) and were still above this threshold 1 year after immunisation. Additionally, neutralising antibody levels were in excess of the protective titres after one dose of our vaccine (i.e. at week 3), indicating that a single dose vaccine for use in livestock is achievable using this antigen–adjuvant combination.

Our finding that lambs with pre-existing neutralising antibodies against epsilon toxin responded less well to vaccination is in line with studies with other vaccines in other species. It is known that pre-existing antibodies to the antigen can inhibit responses to vaccines^37–40^ and it has previously been reported that lambs that acquire maternal antibody from ewes respond poorly to epsilon toxin vaccine.^34^ These pre-existing antibodies might result in clearance of the antigen or the formation of antigen–antibody complexes limiting B cell activation or by physically masking the epitope from B cells.^40^

The vaccine we have devised here would be used in livestock susceptible to enterotoxaemia caused by *C. perfringens* epsilon toxin. It would have a number of advantages over existing vaccines because it does not require detoxification before use. The purity of the antigen and use of an adjuvant such as Montanide ISA 61VG should promote long-term immunity, reducing or eliminating the need for booster immunisations. Alternatively, it may also be possible to use bacteria expressing the toxoid as a bacterin vaccine. The toxoid could also serve as a protein carrier for polysaccharides, which induce protective antibodies against other diseases of livestock.^41–46^ A glycoconjugate would promote T cell responses to the polysaccharide moiety^47^ with increases in the magnitude of the antibody response and the induction of memory responses to the polysaccharide.^48,49^ In addition, the linking of the polysaccharide to the epsilon toxin carrier would allow the vaccine to be used in young animals.^49^ The Y30A-Y196A-A168F protein could be chemically coupled to polysaccharides or it could be further modified to serve as an acceptor for recombinant glycoconjugates generated by exploiting the naturally occurring glycosylation systems in bacteria.^50,51^

## Methods

### Expression and purification of recombinant epsilon toxin mutants

The *etxD* gene, encoding epsilon prototoxin *D* from *C. perfringens* type D strain NCTC 8346, was cloned into the expression vector pET-26b(+) (Merck, Darmstadt, Germany) with a N-terminal PelB leader peptide in place of the 13-amino-acid N-terminal peptide sequence (residues KEISNTVSNEMSK) and with a C-terminal polyhistidine (6× His) tag to aid affinity purification of recombinant prototoxin.^19^ Amino acid numbering corresponds to prototoxin without the 13-amino-acid N-terminal peptide sequence. Wild-type recombinant epsilon prototoxin was produced and purified under ACDP/ACGM containment level 3 conditions.

Mutations H149A, A168F, F92A, V166A, and V72F (residues flanking the β-octyl-glucoside binding site^19^) were introduced into the Y30A-Y196A mutant^20^ using the QuickChange Lightning Site-Directed Mutagenesis Kit (Agilent Technologies Inc., Santa Clara, USA) according to the manufacturer’s instructions. The presence of the intended mutations was verified by DNA-sequencing (Eurofins Genomics, Germany). The proteins were expressed in *E. coli* Rosetta 2 (DE3) cells (Merck, Darmstadt, Germany) and grown in ZYM-5052 auto-induction medium^52^ supplemented with 50 μg/ml kanamycin and 34 μg/ml chloramphenicol. Cells (100 ml) were grown at 37 °C for 3 h and cultured for a further 24 h at 20 °C, 300 rpm.

For protein purification, cells were harvested by centrifugation, lysed enzymatically using BugBuster™ Protein Extraction Reagent (Merck, Darmstadt, Germany), and Y30A-Y196A and its derivatives were purified by Ni-NTA chromatography columns (GE Healthcare Life Sciences, Little Chalfont, UK) according to the manufacturer’s instructions. For buffer exchange and further sample clean up, prototoxin containing eluate was applied to a PD-10 Desalting Column (GE Healthcare Life Sciences, Little Chalfont, UK) and eluted in 10 mM phosphate buffer, 2.7 mM potassium chloride, 137 mM NaCl, pH 7.4. Protein concentrations were determined using a BCA assay (Fisher Scientific UK Ltd, Loughborough, UK).

For studies in mice, rabbits or sheep, the proteins were treated to remove endotoxin using a commercially available kit containing a high capacity endotoxin removal resin (Pierce™ High Capacity Endotoxin Removal Spin Columns, Thermo Scientific) according to the manufacturer’s instructions. Residual levels of endotoxin were measured using a quantitative chromogenic assay (Pierce LAL Chromogenic Endotoxin Quantitation Kit, Thermo Scientific).

### Trypsin activation

Purified recombinant epsilon prototoxin and its derivatives were activated with trypsin, TPCK (l-1-tosylamide-2-phenylethyl chloromethyl ketone) treated, from bovine pancreas (Sigma-Aldrich Company Ltd, Gillingham, UK), which removes the C-terminal peptide sequence. Trypsin was prepared in phosphate-buffered saline (PBS) and added to recombinant prototoxin at 1:100 (w/w) ratio and incubated at room temperature for 1 h. Protease Inhibitor Cocktail, EDTA free (Fisher Scientific UK Ltd, Loughborough, UK), was added to the digest to inhibit trypsin in the samples. Removal of the C-terminal peptide sequence was assessed by sodium dodecyl sulfate-polyacrylamide gel electrophoresis (SDS-PAGE).

### SDS-PAGE analyses

Protein purity was analysed by SDS-PAGE on 4–12% Bis-Tris NuPAGE gels (Invitrogen Ltd, Paisley, UK) using XCell SureLock™ Mini-Cell Electrophoresis System (Invitrogen Ltd, Paisley, UK) and NuPAGE MES SDS running buffer (Invitrogen Ltd, Paisley, UK). All samples were heated prior to loading at 70 °C for 10 min in NuPAGE LDS sample buffer (Invitrogen Ltd, Paisley, UK). Gels were typically run at 200 V for 45 min. After electrophoretic separation, proteins were visualised by SimplyBlue staining (Invitrogen Ltd, Paisley, UK). The Perfect Protein molecular weight standard (Merck, Darmstadt, Germany) was used as marker.

### Thermostability assay

Thermostability was assessed by mixing purified protein (0.25 mg/ml) with 240× SYPRO Orange protein gel stain (Sigma-Aldrich Company Ltd, UK). Fluorescence was monitored using a StepOnePlus quantitative PCR machine (Applied Biosystems, USA) with a 1% thermal gradient from 25 to 99 °C. The fluorescence data obtained was analysed using the Protein Thermal Shift Software (Applied Biosystems, USA) to calculate the *T*_m_ using the Boltzmann method. All measurements were performed in duplicate.

### Construction of CHO stable cell line expressing human or sheep MAL

The *hMAL* gene (NCBI reference NP_002362.1) or the *sMAL* gene (NCBI reference XP_004006224.1) were synthesised (GeneArt, Thermo Fisher Scientific) with the addition of a 5′ *Hin*dIII site and a 3′ *Bam*HI site and each cloned into pEF1αAcGFP-N1 expression vector (Clontech). After verification by sequencing, the plasmids were transfected into CHO cells using Turbofect transfection reagent (Thermo Fisher Scientific) according to the manufacturer’s instructions. Transfected cells were selected in media containing 400 μg/ml G418 for 3 weeks to create a clonal pool and clonal populations expressing hMAL or sMAL were selected and expanded. Clones were then analysed under a fluorescence microscope (Olympus X81) to confirm membrane-associated MAL-GFP expression.

### Cell culture

MDCK.2 cells (ATCC-LGC Standards, Teddington, UK) were routinely cultured in Dulbecco’s modified Eagle’s medium/Ham’s F12 (DMEM/F12) medium (Life Technologies) supplemented with 10% foetal bovine serum (PAA, Pasching, Austria) at 37 °C in a humidified atmosphere of 95% air/5% CO_2_. The culture medium was replaced every 2–3 days. Cells were routinely detached by incubation in trypsin/EDTA and split as appropriate (typically 1:6 dilutions).

Chinese hamster ovary (CHO) cells or CHO cells expressing green fluorescent protein (GFP)-tagged hMAL or sMAL were routinely cultured in Dulbecco’s modified Eagle’s medium/Ham’s F12 (DMEM/F12) medium (Life Technologies) supplemented with 10% (v/v) foetal bovine serum at 37 °C in a humidified atmosphere of 95% air/5% CO_2_. The culture medium was replaced every 2–3 days. Cells were routinely detached by incubation in trypsin/EDTA and split as appropriate (typically 1:6 dilutions).

### Cytotoxicity assay

The cytotoxic activity of trypsin-activated toxins toward MDCK.2 or CHO cells expressing MAL was determined by measuring the amount of lactate dehydrogenase released from the cytosol of lysed cells into the cell culture medium using the CytoTox 96 Non-radioactive Cytotoxicity Assay Kit (Promega UK, Southampton, UK) according to the manufacturer’s protocol. In brief, a two-fold dilution series of each activated toxin (ranging from 10 μM to 0.15 nM) was prepared in PBS and added to cells seeded into 96-well plate (3 × 10^4^ cells/well). Following incubation at 37 °C for 3 h, the cell culture medium (50 μl) was harvested from cell monolayers, transferred to a fresh 96-well enzymatic assay plate and 50 μl of reconstituted substrate mix was added to each well. The plate was incubated for 30 min at room temperature, protected from light. Absorbance was read at 490 nm using a Model 680 Microplate Reader (Bio-Rad). The absorbance values for each sample were normalised by subtracting the absorbance value obtained for the culture medium from untreated cells. The toxin dose required to kill 50% of the cell monolayer (CT_50_) was determined by nonlinear regression analysis (GraphPad). All experiments were performed in triplicate with three technical replicates each.

### Immunisation of rabbits

Groups of three New Zealand White rabbits were each immunised subcutaneously with 100 µg of Y30A-Y196A, Y30A-Y196A-A168F or Y30A-Y196A-H149A-A168F by Cambridge Research Biochemicals. Cambridge Research Biochemicals complies with the Animals (Scientific Procedures) Act 1986 (ASPA) and all project licences are routinely reviewed by the Animal Welfare and Ethics Review Board (AWERB) and the Home Office. Freund’s Complete Adjuvant was used for the initial immunisation and Freund’s Incomplete Adjuvant was used for four subsequent immunisations given at 14-day intervals. Blood was collected 7 days after booster dose 3 (day 49) and 7 days after booster dose 4 (day 63).

### Immunisation of sheep with Y30A-Y196A-A168F toxoid

Immunisations were carried out by Orygen Antibodies Ltd (Penicuik, Scotland). Orygen Antibodies Ltd complies with the Animals (Scientific Procedures) Act 1986 (ASPA) and all project licences are routinely reviewed by the Animal Welfare and Ethics Review Board (AWERB) and the Home Office. Blackface lambs were reared without vaccination against *C. perfringens* epsilon toxin and tested at intervals for the presence of antibody against epsilon toxin using Western blotting.^9^ Western blots to compare antibody levels in lambs were processed in parallel. When the lambs had been weaned and were 12 months old, one group of five lambs received nothing, one group of six lambs received 200 µg of Y30A-Y196A-A168F toxoid adjuvanted (1:1) with Montanide ISA 61VG (Seppic, Paris, France), and a group of five lambs received 200 µg of the Y30A-Y196A-A168F toxoid adjuvanted with alhydrogel (Sigma-Aldrich, Poole UK; 0.25% w/v final concentration) adjuvant. We also immunised a group of four lambs, which had the highest levels of pre-existing reactivity with epsilon toxin by Western blotting, with 200 µg of the Y30A-Y196A-A168F toxoid mixed with Montanide ISA 61VG adjuvant (1:1). All of the adjuvanted mixtures were given s.c. as 6 × 0.5 ml doses. Three weeks later, the lambs were given a second dose of the adjuvanted protein. Blood samples were taken at the start of the study and at 3, 7, 13, 16, 25, 38 and 52 weeks post immunisation.

To measure the level of neutralising antibodies in sera, we also included dilutions of WHO International Standard *C. perfringens* epsilon horse antitoxin serum (National Institute for Biological Standards and Control, South Mimms, UK), which had known levels of neutralising antibody, expressed as IU/ml. A dilution series of these sera provided a standard curve for the calibration of our assays.

### Competition ELISA assay for measuring neutralising antibodies

A competitive ELISA to measure neutralising antibodies was carried out using a Monoscreen ELISA Kit (BioX Diagnostics, BIO K 222/2) according to the manufacturer’s instructions. Absorbance was read at 450 nm and inhibition was calculated using the following formula:

% Inhibition sample = [(OD_450 nm_ negative sera − OD sample)/OD_450 nm_ negative sera] × 100,

% Inhibition positive = [(OD_450 nm_ negative sera −OD positive sera)/OO_450 nm_ negative sera] × 100.

The test was validated only if the OD negative − OD positive was >0.7 and inhibition of the positive control was >30%. A dilution series of International Standard *C. perfringens* epsilon horse antitoxin serum provided a standard curve for the calibration of our assays, as described above.

### Neutralisation of toxicity towards cell cultures

We used two different assays to measure neutralisation of toxicity by antisera. First, we used an assay, using MDCK cells, that has been shown to correlate with neutralisation in mice.^23–27^ Briefly, two-fold dilutions of test sera or standard antitoxin (National Institute of Biological Standards and Control, 40 IU/ml) were made in Eagle’s minimum essential medium supplemented with 5% (v/v) foetal bovine serum, non-essential amino acids, 2 mM l-glutamine and 50 μg/ml gentamicin across a 96-well plate. Trypsin-activated toxin was diluted in the same media to a final concentration of 3.15 µg/ml and an equal volume was added to the wells, followed by incubation at room temperature for 1 h. We added 5 × 10^3^ MDCK.2 cells to each well and the plates were incubated at 37 °C in 95% air/5% CO_2_ for 72 h. Cell viability was assessed using the CellTiter 96 Non-radioactive Cell proliferation assay or MTT assay (Promega).

We also tested the ability of the sera to neutralise toxicity towards CHO cells expressing sheep MAL. These cells were seeded at 3 × 10^4^ cells per well of a 96-well plate and left to adhere overnight. Sheep sera from neat and a standard epsilon antitoxin from 40 IU/ml were diluted in a series of doubling dilutions in Dulbecco’s phosphate-buffered saline (DPBS) and incubated with an equal volume of trypsin-activated wild-type epsilon toxin (5 × CT_50_) for an hour at room temperature. The CHO-sMAL cells were washed twice with serum-free DMEM/F12 and the toxin:antibody/standard antitoxin mix was then added, along with DPBS-only and toxin-only controls (final concentration 5 × CT_50_), to the cells. Following incubation for 3 h at 37 °C in a humidified atmosphere, the media were replaced with 100 μl of fresh serum-free DMEM/F12 and 10 μl of WST-1 cell proliferation reagent (Abcam). The formazan dye produced by viable cells was quantified by measuring the absorbance at 420 nm after incubation for 1 h at 37 °C in a humidified atmosphere.

### Toxicity in mice

Groups of six female 8-week-old BALB/c mice (17–22 g) were challenged by the i.p. or s.c. route with either non-activated or trypsin-activated protein in 100 μl volumes. The experiments were terminated at 24 h post i.p. challenge or 7 days post s.c. challenge. The studies were performed with the approval of the University of Exeter ethics committee. At appropriate intervals, the animals were assessed for neurological symptoms, changes in appearance or changes in behaviour according to a pre-determined scoring matrix. Animals with a combined score of 5 or more were culled.

### Reporting summary

Further information on research design is available in the Nature Research Reporting Summary linked to this article.

## Supplementary information

Reporting Summary

## Data Availability

The datasets generated during and/or analysed in the current study are available from the corresponding author on reasonable request.

## Supplementary information

**Supplementary Information** accompanies the paper on the *npj Vaccines* website (10.1038/s41541-019-0128-2).
